# Concurrent changes in psychological distress and pain thresholds following brief mindfulness: an exploratory phenotyping study of chronic pain

**DOI:** 10.3389/fpsyg.2026.1759576

**Published:** 2026-05-01

**Authors:** Kento Ueda, Hideaki Hasuo, Junji Nishiyama, Hisaharu Shizuma, Fumie Kato, Kohei Yoshida, Mei Xing, Tetsuya Abe, Kenji Kanbara

**Affiliations:** 1 Department of Psychosomatic and General Internal Medicine, Kansai Medical University, Hirakata, Osaka, Japan; 2 Graduate School of Medicine, Kagawa University, Kagawa, Japan

**Keywords:** chronic pain, brief mindfulness intervention, pressure pain threshold, central sensitization, top-down modulation, clinical phenotyping, precision pain care

## Abstract

**Background:**

Clinical trials of brief mindfulness interventions (BMIs) for chronic pain frequently report small average effect sizes, which may mask substantial heterogeneity in treatment responses. Rather than testing general efficacy, this exploratory study investigated whether a BMI is associated with concurrently coupled psychological and sensory improvements within individuals, potentially reflecting top-down nociceptive modulation.

**Methods:**

Fifty female outpatients with chronic pain completed a 2-week BMI. Psychological distress [pain catastrophizing scale (PCS) and hospital anxiety and depression scale (HADS)] and objective sensory markers of central sensitization [pressure pain threshold (PPT)] were assessed pre- and post-intervention. To explore treatment response heterogeneity, multivariate analyses—including principal component analysis (PCA) and cluster analysis—were conducted using individual change scores.

**Results:**

At the group level, the BMI did not produce statistically significant pre-post changes. However, exploratory multivariate analyses revealed strong intra-individual coupling of responses. PCA extracted a single dimension, “simultaneous psychological and sensory improvement,” suggesting that reductions in catastrophizing, anxiety, and depression co-varied concurrently with increases in PPT. Furthermore, cluster analysis identified a highly responsive subgroup (“high psychological improvement”) that exhibited significant concurrent increases in PPT, potentially representing a distinct clinical phenotype.

**Conclusion:**

The true impact of BMIs in chronic pain may lie not in uniform group-level efficacy, but in the concurrent coupling of psychological and sensory improvements within specific individuals. These findings challenge “one-size-fits-all” approaches, highlighting the critical need for mechanism-based patient stratification to advance precision pain care.

## 1 Introduction

Chronic pain is a widespread and debilitating condition affecting approximately 15%−20% of adults, leading to a significant reduction in quality of life, impaired daily functioning, and increased healthcare utilization (Breivik et al., 2006; Nakamura et al., 2011). Contemporary models emphasize its multidimensional nature as a biopsychosocial state, wherein complex interactions among sensory, cognitive, and emotional domains shape the pain experience (Gatchel et al., 2007). Psychological factors, particularly pain catastrophizing, anxiety, and depression, play a central role in the amplification and chronification of pain (Meints and Edwards, 2018). Importantly, these psychological factors not only increase subjective distress but are also closely associated with neurophysiological central sensitization (Meints et al., 2019). Central sensitization refers to the amplification of nociceptive signals within the central nervous system and is objectively evaluated as a reduction in pressure pain threshold (PPT) at non-painful sites (Sjörs et al., 2011; Neziri et al., 2012; Den Bandt et al., 2019). Therefore, to understand the true mechanisms of psychological interventions, it is necessary to concurrently evaluate subjective indices and objective, sensory markers such as PPT.

Mindfulness-based interventions (MBIs) have garnered significant attention as non-pharmacological therapies that foster metacognitive awareness and non-judgmental acceptance, thereby comprehensively reducing not only pain-related catastrophizing but also secondary emotional distress, such as anxiety and depression (Bishop et al., 2004; Schütze et al., 2010; Bawa et al., 2015). Crucially, the neural substrates supporting these cognitive and emotional regulation processes are intimately linked to pain modulation mechanisms (Ossipov et al., 2014; Zeidan and Vago, 2016). It has been proposed that the analgesic mechanisms of mindfulness may involve networks of top-down nociceptive modulation and emotion regulation (Zeidan et al., 2011, 2016). Higher-order cortical networks, including the prefrontal cortex, are associated with the regulation of affective responses and are simultaneously thought to contribute to the modulation of nociceptive signals (Nakajima et al., 2019; Kummer et al., 2020). Therefore, if mindfulness influences central pain processing and emotion regulation via this top-down modulation, it is theoretically predicted that reductions in catastrophizing, anxiety, and depression (psychological improvement) and increases in PPT (sensory improvement) should not occur independently but rather co-vary within individuals.

In recent years, the need for more versatile and accessible approaches has resulted in a notable shift toward brief mindfulness interventions (BMIs), which are programs shortened to less than 2 weeks (Howarth et al., 2019). To date, many clinical trials examining the efficacy of BMIs have reported small average effect sizes at the group level (Howarth et al., 2019; McClintock et al., 2019). However, rather than indicating an ineffective intervention, this likely reflects an oversight of treatment response heterogeneity (substantial inter-individual variability). As the field advances toward the personalization of pain care, research must shift its focus from questioning the “average efficacy” of an intervention to “mechanism-based stratification”—identifying the characteristics of patients who are most likely to show improvement (Bellomo et al., 2020; Butera et al., 2021). Identifying patterns of concurrent psychological and sensory improvement could lead to the discovery of a “clinical phenotype” that is highly responsive to mindfulness.

Given this background, the objective of the present study is not to confirm the general efficacy of BMIs. Rather, it is an exploratory, hypothesis-generating study designed to test the theoretical prediction that mindfulness induces coupled psychological and sensory changes within individuals. By simultaneously evaluating the patterns of change in psychological indices and objective sensory measures (PPT) using multivariate analyses, we aim to elucidate the heterogeneity of treatment responses in patients with chronic pain and propose new hypotheses for future personalized pain management.

## 2 Materials and methods

### 2.1 Participants

This study targeted adult female outpatients with chronic pain. Participants were recruited from a single university hospital in Western Japan between September 2020 and December 2024. Patients diagnosed by a physician based on the International Association for the Study of Pain (IASP) classification of chronic pain were eligible (Treede et al., 2019; Raja et al., 2020). Inclusion criteria were patients aged ≥20 years and having chronic pain lasting for more than 3 months. This study focused exclusively on a female sample. As sex differences are known to exist in pain experience and PPT (Chesterton et al., 2003; Fillingim et al., 2009), standardizing the sex of the sample was considered to eliminate data variability arising from sex differences and allow for a more direct assessment of the intervention's effects. Exclusion criteria included patients with a major psychiatric disorder, cognitive impairment, pain in the forearm (to allow for PPT measurement on a non-painful site), or prior formal training in mindfulness-based programs or meditation.

### 2.2 Study design

This was a single-center, open-label, exploratory, single-arm pre-post comparison prospective clinical study. All assessments and interventions were conducted in a single room at the university hospital affiliated with the principal investigator. Those who participated in the study until the end were given a 3,000-yen gift card as a reward. This study was registered with the University Hospital Medical Information Network Clinical Trials Registry, UMIN000049984. Registered on January 7, 2023 (retrospectively registered). The planning, conduct, and reporting of this study adhere to the transparent reporting of evaluations with nonrandomized designs (TREND) statement checklist for nonrandomized controlled trials.

### 2.3 Ethics statement

The study received approval from the Medical Ethics Committee of Kansai Medical University on March 6, 2019 (reference number: 2019269). The procedures performed in this study were in accordance with the Declaration of Helsinki (as revised in 2013). Written informed consent was obtained from all the participants.

### 2.4 Interventions

Participants underwent a 2-week BMI. The intervention was administered by a clinical psychologist certified in the MBSR program and involved two 40-min, weekly, one-on-one sessions. The first session included psychoeducation on mindfulness and chronic pain, instructions on how to practice mindfulness breathing meditation, a practice period, and a question-and-answer session. Participants were instructed to perform daily 20-min audio-guided home practice of mindfulness breathing meditation. The second session involved a review of their practice and another question-and-answer period. Breathing meditation, which is the foundation of MBIs, is a focused attention meditation that cultivates attentional control and calm judgment by maintaining focus on the sensations of breathing and gently returning the attention whenever it wanders (Lutz et al., 2008). Adherence to the home practice was tracked using self-reported practice diaries.

### 2.5 Outcome measures

Assessments were conducted at baseline and after the 2-week intervention. The primary outcomes were as follows:

- Pressure-pain threshold (PPT): PPT is measured at a non-painful site on the dominant forearm using a digital algometer (Digital Force Gauges RZ Series; AIKOH, Osaka, Japan) fixed to a motorized stand (MODEL-2257, AIKOH, Osaka, Japan) at a rate of 50 kPa/s. The measurement was taken twice, and the average was used (Martel et al., 2013; Corrêa et al., 2015). Pressure algometry is considered a reliable method [intraclass correlation coefficient (ICC) = 0.74–0.89] (Nussbaum and Downes, 1998). Decreased PPT at a non-affected site in patients with chronic pain has been reportedly associated with central sensitization (Sjörs et al., 2011).
- Pain catastrophizing scale (PCS): PCS is a 13-item questionnaire (score range 0–52) assessing pain catastrophizing (Sullivan et al., 1995). It comprises three subscales: rumination, magnification, and helplessness. High reliability has been confirmed (α = 0.87) (Sakano, 2007).
- Hospital anxiety and depression scale (HADS): HADS is a 14-item measuring scale assessing symptoms of anxiety and depression. It has separate subscales for Anxiety (HADS-A; score range 0–21) and depression (HADS-D; score range 0–21) (Zigmond and Snaith, 1983). High reliability has been confirmed for each subscale (HADS-A: α = 0.68–0.93; HADS-D: α = 0.67–0.90) (Bjelland et al., 2002).
- Subjective pain intensity: a 100-mm visual analog scale (VAS) with endpoints labeled “no pain” and “worst imaginable pain.” It is frequently used to assess pain intensity (Ferreira-Valente et al., 2011).

### 2.6 Sample size

The sample size for this study was calculated for an uncontrolled before-and-after design with the change in the PPT as the primary outcome. The calculation was based on the results of a similar prior study by Ahn et al. (2019). To ensure a conservative estimate, data on the change in the PPT at the lateral knee, which had a smaller effect size, were used. In that study, the intervention group exhibited a mean change of 0.75 kgf with a standard deviation (SD) of 1.29 kgf. This yielded a Cohen's *d* of 0.58 for a paired *t*-test. A power analysis was conducted using G^*^Power software (Ver. 3.1.9.2) (Heinrich-Heine-Universität Düsseldorf, Düsseldorf, Germany). With a significance level (α) of 0.05 (two-tailed) and a statistical power of 0.95, a minimum of 41 participants was required to complete the study. Accounting for a potential dropout rate of 25%, as reported in similar MBI studies (Lymeus et al., 2019), this study aimed to recruit a total of 54 participants.

### 2.7 Statistical analysis

This study was conducted on an intention-to-treat (ITT) population, where data from participants who dropped out during the intervention were included in the final analysis (Detry and Lewis, 2014). For participants who completed the study, their final observed values were used. For those who dropped out, their baseline values were carried forward as the final 2-week assessment values. This conservative imputation method was selected to prevent overestimation of treatment effects commonly associated with dropouts in pain intervention trials. All analyses were performed using IBM SPSS Statistics (Ver. 27.0; IBM Japan Ltd., Minato City, Japan). Normality was assessed using the Shapiro–Wilk test. Pre-post comparisons for each outcome were examined using paired *t*-tests or Wilcoxon signed-rank tests for non-normally distributed data. Effect sizes were calculated as Cohen's *d* (parametric) or *r* (non-parametric). Pearson's correlation coefficients were calculated between the change scores (Δ = post-intervention – pre-intervention).

Based on the theoretical background that mindfulness interventions first induce changes in the cognitive and emotional processes, such as pain catastrophizing, anxiety, and depression (Gard et al., 2012; Zeidan et al., 2012; Day et al., 2015; Lu et al., 2023), *K*-means cluster analysis was used to group participants based on their psychological change scores (Δ-PCS, Δ-HADS-A, Δ-HADS-D) to explore the variability in the response. Differences in Δ-PPT, Δ-VAS, and baseline values between clusters were compared using independent samples *t*-tests. Principal component analysis (PCA) was performed on the Δ scores, and components with eigenvalues greater than 1 were extracted. Differences in the extracted PCA scores between clusters were analyzed using an independent samples *t*-test. The statistical significance level was set at *p* < 0.05 (two-sided).

## 3 Results

### 3.1 Participant characteristics

**Figure 1:**
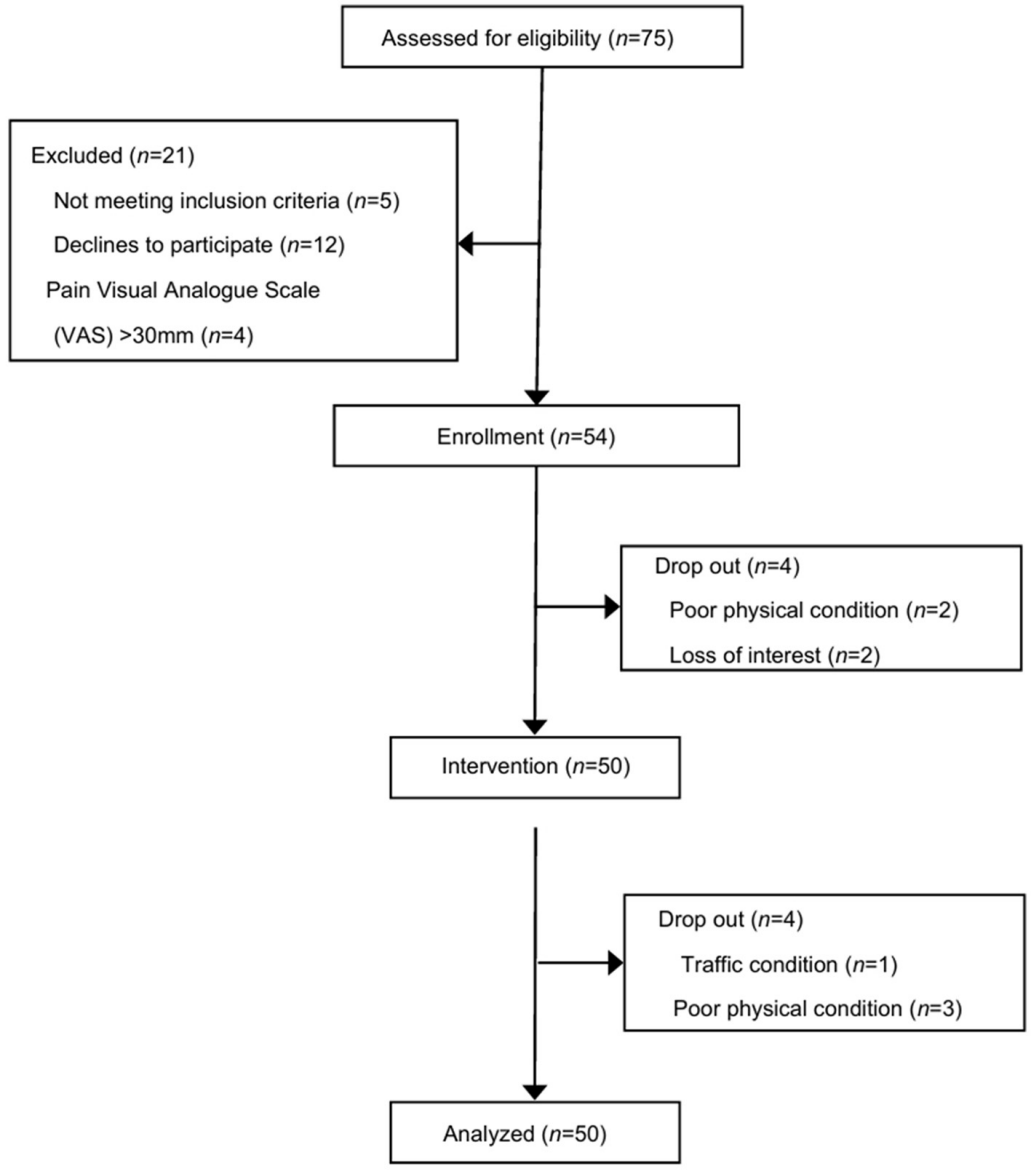
Participant's flow diagram. In the intention-to-treat (ITT) analysis, data for dropouts were carried over from the baseline values.

Of the 75 participants screened, 54 were enrolled, and 50 were included in the statistical analysis (mean age 51.7 ± 12.8 years, mean pain duration 82.5 ± 73.2 months). The flow of participants from screening to statistical analysis is shown in Figure 1. Participant characteristics are presented in Table 1, and the IASP classification of chronic pain for the participants is shown in Table 2. Regarding intervention adherence, there were eight dropouts during the intervention period (completion rate: 85.2%). Among the 46 participants who completed the intervention, the average adherence to homework was 13.4 out of 14 days (95.5%, range: 7–15 days), and the average practice time was 255.0 out of 280 min (91.1%, range: 130–345 min).

**Table 1:** Baseline characteristics of participants (*N* = 50).

Characteristic	Value
Age, years	51.7 ± 12.8
Body mass index, kg/m^2^	21.4 ± 3.8
Education, *n* (%)	
<High school	1 (2%)
High school	19 (38%)
≥University/college	30 (60%)
Marital status, *n* (%)	
Unmarried	18 (36%)
Married	26 (52%)
Divorced/widowed	6 (12%)
Pain duration, *n* (%)	
<12 months	2 (4%)
12 to <60 months	24 (48%)
≥60 months	24 (48%)

Values are presented as mean ± standard deviation or n (%) as indicated.

**Table 2:** Participants' clinical diagnostic classification (*N* = 50).

Chronic pain classifications	Value; *N* (%)
Chronic primary pain	29 (58.00)
Chronic cancer pain	1 (2.00)
Chronic postsurgical and posttraumatic pain	5 (10.00)
Chronic neuropathic pain	5 (10.00)
Chronic headache and orofacial pain	4 (8.00)
Chronic visceral pain	2 (4.00)
Chronic musculoskeletal pain	4 (8.00)
Pain duration (months)	
<12 months	2 (4.00)
12 to <60 months	24 (48.00)
≥60 months	24 (48.00)

The diagnosis and classification of chronic pain is based on the clinical diagnostic classification provided by the International Association for the Study of Pain (IASP).

### 3.2 Pre–post comparison

Following the 2-week BMI, no statistically significant changes were observed in the primary psychological and sensory outcome measures at the group level. Specifically, although the PCS total, HADS-anxiety, HADS-depression, and VAS scores decreased by 1.2 ± 5.1 (mean ± SD), 1.7 ± 5.4, 1.3 ± 5.4, and 8.7 ± 15.3 mm, respectively, and PPT increased by 28.4 ± 60.1 kPa, these pre–post changes did not reach statistical significance (Table 3). Only the magnification subscale of the PCS demonstrated a statistically significant change (*p* < 0.05).

**Table 3:** Pre–post changes in outcome measures (*N* = 50).

Outcome measure	Pre-intervention [mean ±SD or median (IQR)]	Post-intervention [mean ±SD or median (IQR)]	Change [mean ±SD or median (IQR)]	*p*-value	Effect size
PPT (kPa)	243.1 ± 135.5	271.5 ± 160.8	28.4 ± 60.1	0.07	*d* = 0.26
PCS				
Total	33.4 ± 10.0	32.2 ± 9.8	−1.2 ± 5.1	0.10	*d* = 0.24
Rumination	15.0 [12.8–18.3]	15.0 [11.0–18.0]	0 [−1.3–2.0]	0.90	*r* = 0.02
Magnification	7.5 [5.0–10.0]	7.0 [4.0–9.0]	−0.5 [−2.0–0.3]	0.04^*^	*r* = 0.29
Helplessness	10.5 [7.0–15.0]	10.0 [7.0–14.0]	−0.5 [−3.0–1.0]	0.06	*r* = 0.27
HADS				
Anxiety	9.7 ± 4.3	8.0 ± 4.9	−1.7 ± 5.4	0.10	*d* = 0.24
Depression	10.0 ± 4.8	8.6 ± 5.3	−1.3 ± 5.4	0.14	*d* = 0.22
VAS (mm)	62.5 ± 17.6	53.8 ± 20.9	−8.7 ± 15.3	0.14	*d* = 0.21

^*^p <0.05.

Values are presented as mean ± SD or median (interquartile range). p-values for normally distributed data (PCS total, HADS, VAS, PPT) are from paired-samples t-tests. Effect sizes are presented as Cohen's d.

PPT, pressure pain threshold; PCS, pain catastrophizing scale; HADS, hospital anxiety and depression scale; VAS, visual analog scale.

### 3.3 Correlation analysis of the changes

Pearson's correlation analysis between the change scores revealed significantly small associations (Table 4). The change in the PCS total scores negatively correlated with the change in the PPT (*r* = −0.32, *p* = 0.02) and positively correlated with the change in the VAS scores (*r* = 0.39, *p* = 0.01). Changes in the HADS-A and HADS-D scores demonstrated significant positive correlations with the change in the PCS total score (HADS-A: *r* = 0.37, *p* = 0.01; HADS-D: *r* = 0.35, *p* = 0.01). Additionally, significant weak negative correlations were observed between the changes in the HADS-A and HADS-D scores and the change in the PPT (HADS-A: *r* = −0.29, *p* = 0.04; HADS-D: *r* = −0.31, *p* = 0.03).

**Table 4:** Correlation between changes in measurement items (*N* = 50).

Variable	Δ-HADS-D	Δ-PCS total	Δ-VAS	Δ-PPT
Δ-HADS-A	0.43^**^	0.37^**^	0.36^**^	−0.29^*^
Δ-HADS-D		0.35^**^	0.36^**^	−0.31^*^
Δ-PCS total			0.39^**^	−0.32^*^
Δ-VAS				−0.38^**^

^*^p <0.05, ^**^p <0.01.

For each measurement item, the difference between the post-intervention value and the pre-intervention value was used as the change amount (Δ).

HADS, hospital anxiety and depression scale; PCS, pain catastrophizing scale; VAS, visual analog scale; PPT, pressure pain threshold.

### 3.4 Cluster analysis

*K*-means cluster analysis, based on the psychological change scores (Δ-PCS total, Δ-HADS-A, Δ-HADS-D), divided participants into two subgroups: a “High Psychological Improvement” group (*n* = 23) and a “low psychological improvement” group (*n* = 27). The high psychological improvement group demonstrated a significantly greater increase in the PPT than the low psychological improvement group (+57.4 ± 127.6 kPa vs. −0.1 ± 65.7 kPa, *p* = 0.046). No significant differences were observed in the demographic variables or baseline values between the clusters (Table 5).

**Table 5:** Comparison of changes in pain outcomes and baseline characteristics between “high improvement” and “low improvement” clusters identified by *K*-means analysis.

Score	High improvement (*n* = 23)	Low Improvement (*n* = 27)	*p*-value
	Mean ±SD	Mean ±SD	
Δ-PPT (kPa)	57.4 ± 127.6	−0.1 ± 65.7	0.046^*^
Δ-VAS (mm)	−7.43 ± 18.3	0.4 ± 11.1	0.08
Age (years)	51.0 ± 13.1	52.2 ± 12.7	0.76
Body mass index (kg/m^2^)	20.8 ± 3.5	21.8 ± 4.0	0.32
Pain duration (months)	65.7 ± 62.0	96.8 ± 79.9	0.13
PCS			
Total	35.7 ± 10.2	31.6 ± 9.5	0.15
Rumination	15.7 ± 3.9	14.1 ± 3.5	0.14
Magnification	7.6 ± 3.2	7.1 ± 2.4	0.50
Helplessness	12.4 ± 4.6	10.4 ± 4.9	0.15
HADS			
Anxiety	10.1 ± 4.5	9.4 ± 4.2	0.57
Depression	11.3 ± 5.1	8.9 ± 4.4	0.08
VAS (mm)	64.7 ± 15.3	60.7 ± 19.4	0.42
PPT (kPa)	220.3 ± 104.4	262.5 ± 156.6	0.28

^*^p <0.05.

Clusters were classified based on psychological change scores (Δ-PCS total, Δ-HADS-anxiety, Δ-HADS-depression). Independent sample t-tests were used for comparisons.

SD, standard deviation; PPT, pressure-pain threshold; VAS, visual analog scale; PCS, pain catastrophizing scale; HADS, hospital anxiety and depression scale.

### 3.5 PCA

PCA of the change scores extracted a single component reflecting simultaneous improvement across the pain and psychological domains. This component, “simultaneous psychological and sensory improvement,” had an eigenvalue of 2.43, indicating 48.60% of the variance. It was characterized by a negative loading from PPT and positive loadings from PCS total, HADS-A, HADS-D, and VAS (Table 6). An independent samples *t*-test revealed a significant difference in the scores for this component between the “high psychological improvement” and “low psychological improvement” clusters (*p* = 0.00).

**Table 6:** Principal component analysis of outcome change scores (*N* = 50).

Variable (change score)	Component 1
ΔPPT	−0.53
ΔPCS	0.90
ΔHADS-anxiety	0.60
ΔHADS-depression	0.60
ΔVAS	0.62
Eigenvalue	2.43
% of variance explained	48.60%

The single component reflects a pattern of simultaneous improvement in psychological distress (PCS, HADS), pain intensity (VAS), and sensory sensitivity (PPT).

Extraction method: PCA. Rotation was not applied as only one component was extracted.

PPT, pressure-pain threshold; PCS, pain catastrophizing scale; HADS, hospital anxiety and depression scale; VAS, visual analog scale.

## 4 Discussion

The primary finding of this exploratory single-arm pre-post comparison prospective clinical study is that although a 2-week BMI did not produce statistically significant mean changes at the group level in female adults with chronic pain, tracking the individual-level changes revealed a clear pattern of strong coupling between psychological and sensory improvements. Although a significant difference was observed solely in the magnification subscale of the PCS, this requires cautious interpretation as no correction for multiple comparisons was applied. Multivariate analyses focusing on individual-level change scores demonstrated that psychological improvements—such as reductions in catastrophizing, anxiety, and depression—strongly co-vary with increases in PPT, an objective marker of central sensitization, concurrently within specific patients.

The extraction of a single principal component of “simultaneous psychological and sensory improvement” from all the change scores indicates that these improvements do not occur independently but are linked through a common underlying mechanism. Although direct causal relationships or directionality cannot be determined from correlations between change scores, this coupling aligns with recent top-down models of nociceptive modulation. This mechanism may involve a reduction in catastrophic thinking through enhanced metacognitive awareness and improved emotional regulation abilities cultivated by mindfulness practice (Gard et al., 2012; Zeidan et al., 2012; Day et al., 2015; Lu et al., 2023). These cognitive and emotional processes, which reduce psychological distress, including anxiety and depression, alongside pain-related catastrophizing, are thought to be associated with the activity of higher-order cortical networks, including the prefrontal cortex (Zeidan et al., 2011, 2016). As this network may simultaneously influence the top-down regulation of emotion and the modulation of nociceptive signals, it supports the hypothesis that psychological improvement and objective increases in PPT manifest concurrently within individuals.

Indeed, our cluster analysis clearly identified a “high psychological improvement group” that demonstrated a significant increase in the objective sensory measure of PPT compared with the “low psychological improvement group.” Given the small sample size and the exploratory nature of the analysis using change scores, further validation is required to assess the stability and generalizability of this cluster. However, this subgroup exhibiting a “coupled psychological and sensory response” may represent a measurable “clinical phenotype” for which mindfulness may be effective. This finding clearly illustrates the limitations of a one-size-fits-all approach to chronic pain management and suggests the importance of “mechanism-based stratification” based on intervention responsiveness (Bellomo et al., 2020; Butera et al., 2021).

**Figure 2:**
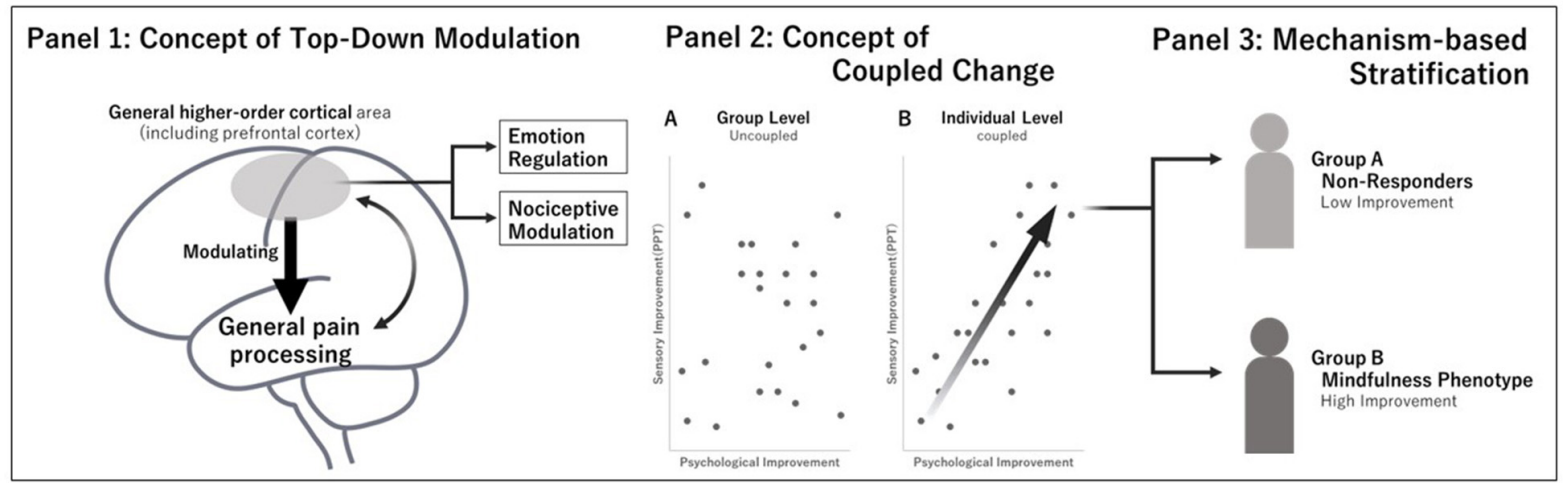
Conceptual model of top-down modulation, coupled psychological and sensory responses, and mechanism-based patient stratification. **Panel 1:** top-down modulation. Mindfulness practice is hypothesized to engage higher-order cortical networks, potentially influencing emotion regulation and nociceptive signals simultaneously. **Panel 2:** coupled changes. Scatter plots contrasting uncoupled group-level averages **(left)** with intra-individual coupling **(right)**. In the coupled response, psychological improvements (reduced PCS/HADS) concurrently co-vary with objective sensory improvements (increased PPT). **Panel 3:** mechanism-based stratification. Based on specific response patterns, patients can be stratified into non-responders and a highly responsive clinical phenotype, highlighting the need for personalized pain care.

The lack of significant group-level findings is consistent with the modest effect sizes reported in meta-analyses of BMIs (Howarth et al., 2019; McClintock et al., 2019). Upon integrating these findings, it is plausible that the failure to detect a group-level effect in this study was not attributable to ineffective interventions but to the inability of traditional statistical approaches—assuming a homogeneous effect—to capture heterogeneous treatment responses characterized by large individual differences. Rather, it may reflect that mechanistic changes (i.e., coupling) are concentrated within a subset of patients possessing a specific clinical phenotype (a dilution effect). Traditional approaches that average the entire sample risk overlooking dynamic, intra-individual changes occurring within patients (Figure 2).

### 4.1 Limitations

Some important limitations of this study should be acknowledged.

First and foremost, the uncontrolled before-and-after design precludes causal inference, and the observed changes could be influenced by non-specific factors such as the placebo effect, expectation, regression to the mean, or the passage of time. Consequently, the findings must be interpreted strictly as exploratory and hypothesis-generating, rather than as a confirmatory test of intervention efficacy.

Second, to handle missing data from dropouts, we utilized an ITT approach with baseline observation carried forward. Although this conservative imputation method was deliberately chosen to prevent overestimation of treatment effects commonly seen in pain intervention trials, it does not reflect the actual trajectories of change for these individuals and carries the inherent risk of underestimating the true treatment effect and variance within the sample.

Third, the multivariate analyses, including correlations and cluster analysis, are exploratory in nature. Although the correlations among change scores suggest a strong coupling of psychological and sensory improvements, they do not establish causality or directionality (i.e., which change preceded the other). Furthermore, the clusters identified using change scores in a relatively small sample size (*N* = 50) may be statistically unstable. To confirm the generalizability and reliability of the proposed “clinical phenotype,” validation in larger, independent cohorts is essential.

Fourth, the clinical trial registration on the UMIN registry (UMIN000049984) was completed retrospectively. This occurred as a result of an administrative oversight; the research team mistakenly believed the final submission step had been completed prior to the study's commencement and only realized the process was pending after enrollment had begun, at which point it was immediately finalized. It is important to emphasize that the study protocol received rigorous prior approval from the Institutional Review Board (March 6, 2019) and was conducted strictly without deviation, thereby preserving the study's ethical validity and integrity. Nevertheless, we acknowledge that the lack of prospective registration impacts the transparency of the trial.

Finally, participants were recruited from a single department at a single institution, which may have introduced selection bias. Adherence to home practice was self-reported, which may limit the generalizability of the findings to broader or more diverse chronic pain populations. As this study was conducted exclusively with a female sample, the results should be interpreted with caution, and further investigation in male participants is necessary for generalization.

Future research should address these limitations by conducting a randomized controlled trial with a larger sample size to rigorously test the hypotheses generated by this study.

## 5 Conclusions

The exploratory analysis in this study suggests that a 2-week BMI may be associated with concurrently coupled psychological and sensory improvements in specific individuals with chronic pain, rather than producing uniform group-level effects. This coupled response may reflect top-down modulation, suggesting a highly responsive clinical phenotype. These findings challenge “one-size-fits-all” approaches and highlight the critical need for mechanism-based patient stratification to advance precision pain care.

## Data Availability

The raw data supporting the conclusions of this article will be made available by the authors, without undue reservation.
